# Age-specific considerations in aetiology of paediatric out-of-hospital cardiac arrest

**DOI:** 10.1186/s13049-025-01385-4

**Published:** 2025-05-01

**Authors:** Deliah Bockemuehl, Alexander Fuchs, Roland Albrecht, Robert Greif, Martin Mueller, Urs Pietsch

**Affiliations:** 1 Department of Anaesthesiology, Emergency and Pain Medicine, Cantonal Hospital St. Gallen, St. Gallen, Switzerland; 2 Department of Anaesthesiology, Emergency and Pain Medicine, Basel University Hospital, University of Basel, Spitalstrasse 21, Basel, 4031 Switzerland; 3 Department of Anaesthesiology and Pain Medicine, Inselspital, Bern University Hospital, University of Bern, Bern, Switzerland; 4 Swiss Air-Ambulance (Rega), Zurich, Switzerland; 5 Department of Perioperative Intensive Care Medicine, Cantonal Hospital St. Gallen, St. Gallen, Switzerland; 6 Department of Emergency Medicine, Bern University Hospital, Inselspital, University of Bern, Bern, Switzerland; 7 Medical Faculty, University of Bern, Bern, Switzerland; 8 European Resuscitation Council (ERC) Research NET, Niel, Belgium

Dear Editor,

The recent advancements in paediatric out-of-hospital cardiac arrest (OHCA) management underscore the importance of understanding age-specific aetiologies and their influence on neurological outcomes. Based on our analysis of 296 paediatric patients up to 16 years of age treated by helicopter emergency medical services (HEMS) between 01–01–2011 and 31–12–2021, we noted that favourable neurologicaloutcomes, defined by a Cerebral Performance Category (CPC) score of 1 or 2 at 30 days post-arrest, were achieved in 18.9% of cases [1]. By examining age-specific trends (Fig. 1), we aim to gain a deeper understanding of the aetiology and to highlight critical aspects for improving favourable neurological outcome [1, 2].

**Fig. 1:**
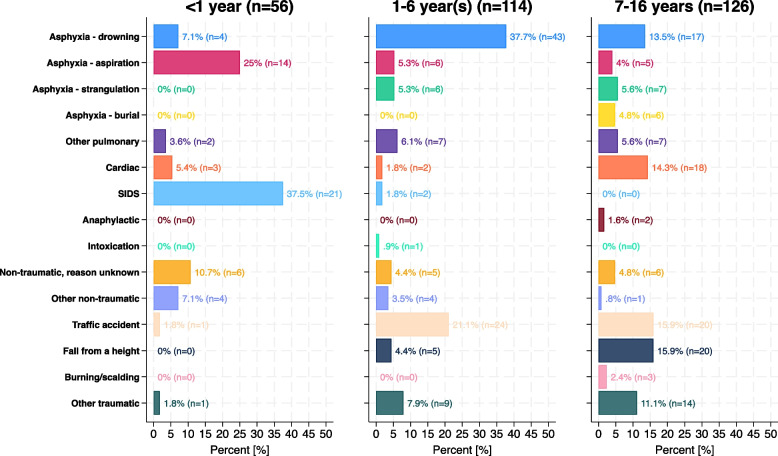
Age specific categorization of the aetiology of paediatric out of hospital cardiac arrest

## Cardiac vs. non-cardiac aetiology across ages

In our cohort, 23 cases of paediatric OHCA were due to cardiac causes, with a median patient age of 10 years. Among these, 65% had a known cardiac comorbidity, and immediate bystander cardiopulmonary resuscitation (CPR) was often initiated, suggesting greater awareness among caregivers trained for critical events. Key cardiac factors included congenital heart defects (e.g., hypoplastic left heart syndrome), acquired cardiomyopathies and arrhythmias (e.g., Long QT syndrome) [3]. Non-cardiac causes, however, accounted for a significant portion (*n* = 233, 78%) of arrests and varied in origin across age groups, underscoring the need for age-targeted strategies. These non-cardiac aetiologies included respiratory insufficiency (mostly due to asphyxia as well as respiratory infections), trauma and metabolic derangements.

## *Infants (*< *1 Year): high risk of hypoxia-related arrests*

Infants demonstrate a unique vulnerability to hypoxia-related arrests, primarily due to physiological factors such as higher metabolic rates, lower functional residual capacity and immature cardiovascular response [4]. In this age group, 89.3% of cases were non-traumatic, with sudden infant death syndrome (SIDS) and bronchopulmonary aspiration accounting for 37.5% and 25% of cases, respectively. The diagnosis of SIDS is made when there is no explanation for cardiac arrest found after thorough investigation. Pathophysiological mechanisms remain mostly unclear, while several risk factors were identified such as prone sleeping, over-heating, smoke exposure and infection [5]. Infants with return of spontaneous circulation (ROSC) upon arrival of HEMS had significantly improved neurological outcomes (*p* < 0.05). This age group had a high incidence (53.6%) of asystole as the initial rhythm with no prevalence of favourable neurological outcome, emphasizing the importance of rapid intervention to restore oxygenation and minimize neurological damage (Table 1).

**Table 1:** Detailed baseline characteristics of patient group < 1 year old. Adjusted to 30-day favourable neurological outcome defined as cerebral performance categories (CPC) 1 and 2. Data presented in n (%)

Variables	Total		Favor. neurol. outc. (docum.)				
			**No (CPC > 2)**		**Yes (CPC 1/2)**		***P*-value**
**Patient & mission characteristics**							
Age group							
< 1y	56	(100.0)	45	(100.0)	11	(100.0)	-
Late/night mission (20:00–07:59)	10	(17.9)	10	(22.2)	0	(0.0)	0.085
**Activity**							
Transport							
Car	1	(1.8)	1	(2.2)	0	(0.0)	0.618
Watersport							
Swimming	1	(1.8)	1	(2.2)	0	(0.0)	0.618
Other							
In and around the house	44	(78.6)	38	(84.4)	6	(54.5)	
No activity or unknown	10	(17.9)	5	(11.1)	5	(45.5)	0.026
**Aetiology detail**							
**Non-traumatic**	54	(96.4)	43	(95.6)	11	(100.0)	0.476
Asphyxia—total	18	(32.1)	15	(33.3)	3	(27.3)	0.700
Asphyxia—drowning	4	(7.1)	4	(8.9)	0	(0.0)	0.305
Asphyxia—aspiration	14	(25.0)	11	(24.4)	3	(27.3)	0.846
Asphyxia—strangulation	0	[0.0]	0	[0.0]	0	[0.0]	-
Asphyxia—burial	0	[0.0]	0	[0.0]	0	[0.0]	-
Other pulmonary	2	(3.6)	0	(0.0)	2	(18.2)	0.004
Cardiac	3	(5.4)	2	(4.4)	1	(9.1)	0.540
SIDS	21	(37.5)	21	(46.7)	0	(0.0)	0.004
Anaphylactic	0	[0.0]	0	[0.0]	0	[0.0]	-
Intoxication	0	[0.0]	0	[0.0]	0	[0.0]	-
Non-traumatic, reason unknown	6	(10.7)	3	(6.7)	3	(27.3)	0.048
Other non-traumatic	4	(7.1)	2	(4.4)	2	(18.2)	0.113
**Traumatic**	2	(3.6)	2	(4.4)	0	(0.0)	0.476
Traffic accident	1	(1.8)	1	(2.2)	0	(0.0)	0.618
Fall from a height	0	[0.0]	0	[0.0]	0	[0.0]	-
Burning/scalding	0	[0.0]	0	[0.0]	0	[0.0]	-
Other traumatic	1	(1.8)	1	(2.2)	0	(0.0)	0.618
**First rhythm**							
**Non-shockable**	54	(96.4)	43	(95.6)	11	(100.0)	0.476
Pulseless Electrical Activity	8	(14.3)	6	(13.3)	2	(18.2)	0.680
Asystole	31	(55.4)	31	(68.9)	0	(0.0)	< 0.001
**Shockable initial rhythm**	2	(3.6)	2	(4.4)	0	(0.0)	0.476
Pulseless Ventricular Tachycardia	0	[0.0]	0	[0.0]	0	[0.0]	-
Pulseless Ventricular Fibrillation	2	(3.6)	2	(4.4)	0	(0.0)	0.476
Normal Sinus rhythm / ROSC	5	(8.9)	0	(0.0)	5	(45.5)	< 0.001
Unknown	10	(17.9)	6	(13.3)	4	(36.4)	0.074
No measures taken (obviously dead)	0	[0.0]	0	[0.0]	0	[0.0]	-
**Injuries**							
Traumatic brain injury	2	(3.6)	2	(4.4)	0	(0.0)	0.476
Chest trauma	0	[0.0]	0	[0.0]	0	[0.0]	-
Abdominal trauma	0	[0.0]	0	[0.0]	0	[0.0]	-
Pelvic trauma	0	[0.0]	0	[0.0]	0	[0.0]	-
Upper extremity trauma	0	[0.0]	0	[0.0]	0	[0.0]	-
Lower extremity trauma	0	[0.0]	0	[0.0]	0	[0.0]	-
**Outcome**							
Favor. neurol. outcome, 30d (CPC 1/2)	11	(19.6)	0	(0.0)	11	(100.0)	< 0.001

Depending on normality testing (Shapiro Wilk) median (IQR) respectively mean (SD) are shown for continuous variables, *p*-values obtained by Wilcoxon rank sum test respectively unpaired T-test. Categorical variables are shown with number (%) in each category, *p*-values obtained by Chi-squared test

*Abbreviations*: *CPC* Cerebral Performance Category, *ROSC* Return of Spontaneous Circulation, *SIDS* Sudden Infant Death Syndrome

## Preschoolers (1–6 Years): drowning as the leading cause

In preschool-aged children, drowning was the predominant cause of cardiac arrest, responsible for 37.7% of cases. Many of these incidents occurred in swimming pools, where immediate bystander reactions, mainly through lifeguards, contributed to improved survival and neurological outcomes (*p* < 0.001, Table 2). This age group benefits substantially from preventive measures and caregiver education on basic life support skills [6]. Traumatic events, primarily from traffic accidents, accounted for 33.4% of cases. The lower frequency of nighttime missions in preschool-aged children may be attributed to their daytime activity patterns, increased supervision during waking hours, reduced engagement in risky behaviours, and the timing of common incidents such as drowning, which typically occur during daytime periods (Table 2).

**Table 2:** Detailed baseline characteristics of patient group 1–6 years old. Adjusted to 30-day favourable neurological outcome defined as cerebral performance categories (CPC) 1 and 2. Data presented in n (%)

Variables	Total		Favor. neurol. outc. (docum.)				
			**No (CPC > 2)**		**Yes (CPC 1/2)**		***P*-value**
**Patient & mission characteristics**							
Age group							
1-6y	114	(100.0)	86	(100.0)	28	(100.0)	-
Late/night mission (20:00–07:59)	8	(7.0)	8	(9.3)	0	(0.0)	0.094
**Activity**							
Transport							
Car	3	(2.6)	3	(3.5)	0	(0.0)	
Bicycle	1	(0.9)	1	(1.2)	0	(0.0)	
Pedestrian	8	(7.0)	8	(9.3)	0	(0.0)	
Other means of transport	3	(2.6)	3	(3.5)	0	(0.0)	0.229
Watersport							
Swimming	21	(18.4)	7	(8.1)	14	(50.0)	< 0.001
Summersport							
Hiking	5	(4.4)	4	(4.7)	1	(3.6)	0.809
Wintersport							
Skiing/Carving	3	(2.6)	2	(2.3)	1	(3.6)	
Other winter sports	1	(0.9)	1	(1.2)	0	(0.0)	0.798
Work							
In agriculture	1	(0.9)	1	(1.2)	0	(0.0)	0.567
Other							
No other activity	46	(40.4)	30	(34.9)	16	(57.1)	
In and around the house	60	(52.6)	48	(55.8)	12	(42.9)	
Relocation (secondary deployment)	1	(0.9)	1	(1.2)	0	(0.0)	
No activity or unknown	7	(6.1)	7	(8.1)	0	(0.0)	0.118
**Aetiology detail**							
**Non-traumatic**	76	(66.7)	49	(57.0)	27	(96.4)	< 0.001
Asphyxia – total	55	(48.2)	32	(37.2)	23	(82.1)	< 0.001
Asphyxia – drowning	43	(37.7)	24	(27.9)	19	(67.9)	< 0.001
Asphyxia – aspiration	6	(5.3)	5	(5.8)	1	(3.6)	0.644
Asphyxia – strangulation	6	(5.3)	3	(3.5)	3	(10.7)	0.137
Asphyxia – burial	0	[0.0]	0	[0.0]	0	[0.0]	-
Other pulmonary	7	(6.1)	6	(7.0)	1	(3.6)	0.514
Cardiac	2	(1.8)	2	(2.3)	0	(0.0)	0.416
SIDS	2	(1.8)	2	(2.3)	0	(0.0)	0.416
Anaphylactic	0	[0.0]	0	[0.0]	0	[0.0]	-
Intoxication	1	(0.9)	0	(0.0)	1	(3.6)	0.078
Non-traumatic, reason unknown	5	(4.4)	4	(4.7)	1	(3.6)	0.809
Other non-traumatic	4	(3.5)	3	(3.5)	1	(3.6)	0.983
**Traumatic**	38	(33.3)	37	(43.0)	1	(3.6)	< 0.001
Traffic accident	24	(21.1)	24	(27.9)	0	(0.0)	0.002
Fall from a height	5	(4.4)	4	(4.7)	1	(3.6)	0.809
Burning/scalding	0	[0.0]	0	[0.0]	0	[0.0]	-
Other traumatic	9	(7.9)	9	(10.5)	0	(0.0)	0.074
**First rhythm**							
**Non-shockable**	109	(95.6)	81	(94.2)	28	(100.0)	0.192
Pulseless Electrical Activity	18	(15.8)	16	(18.6)	2	(7.1)	0.149
Asystole	57	(50.0)	56	(65.1)	1	(3.6)	< 0.001
**Shockable initial rhythm**	5	(4.4)	5	(5.8)	0	(0.0)	0.192
Pulseless Ventricular Tachycardia	0	[0.0]	0	[0.0]	0	[0.0]	-
Pulseless Ventricular Fibrillation	5	(4.4)	5	(5.8)	0	(0.0)	0.192
Normal Sinus rhythm / ROSC	23	(20.2)	1	(1.2)	22	(78.6)	< 0.001
Unknown	9	(7.9)	6	(7.0)	3	(10.7)	0.524
No measures taken (obviously dead)	2	(1.8)	2	(2.3)	0	(0.0)	0.416
**Injuries**							
Traumatic brain injury	29	(25.4)	28	(32.6)	1	(3.6)	0.002
Chest trauma	10	(8.8)	10	(11.6)	0	(0.0)	0.059
Abdominal trauma	7	(6.1)	7	(8.1)	0	(0.0)	0.119
Pelvic trauma	1	(0.9)	1	(1.2)	0	(0.0)	0.567
Upper extremity trauma	1	(0.9)	1	(1.2)	0	(0.0)	0.567
Lower extremity trauma	4	(3.5)	4	(4.7)	0	(0.0)	0.245
**Outcome**							
Favor. neurol. outcome, 30d (CPC 1/2)	28	(24.6)	0	(0.0)	28	(100.0)	< 0.001

Depending on normality testing (Shapiro Wilk) median (IQR) respectively mean (SD) are shown for continuous variables, *p*-values obtained by Wilcoxon rank sum test respectively unpaired T-test. Categorical variables are shown with number (%) in each category, *p*-values obtained by Chi-squared test

*Abbreviations*: *CPC* Cerebral Performance Category, *ROSC* Return of Spontaneous Circulation, *SIDS* Sudden Infant Death Syndrome

## School-aged and adolescent children (7–16 Years): high incidence of traumatic arrests

For children aged 7–16, traumatic causes—primarily traffic accidents and falls from heights (both 15.9%)—comprised 45.3% of cardiac arrest cases. Non-traumatic causes, such as acquired cardiomyopathies and arrhythmias, were also prevalent (54.7%). Unlike in younger age groups, trauma-related OHCAs were associated with lower rates of favourable neurological outcomes, reflecting the challenge of managing traumatic injuries before emergency medical service (EMS) arrival. Standardized guidelines for paediatric traumatic cardiac arrest (TCA) remain limited, underscoring a gap in age-specific treatment strategies for this group. Although age differences alone were not statistically significant in predicting favourable outcomes, traumatic aetiologies notably influenced survival rates [7] (Table 3).

**Table 3:** Detailed baseline characteristics of patient group 7–16 years old. Adjusted to 30-day favourable neurological outcome defined as cerebral performance categories (CPC) 1 and 2. Data presented in n (%)

Variables	Total		Favor. neurol. outc. (docum.)				
			**No (CPC > 2)**		**Yes (CPC 1/2)**		***P*-value**
**Patient & mission characteristics**							
Age group							
7-16y	126	(100.0)	109	(100.0)	17	(100.0)	-
Late/night mission (20:00–07:59)	27	(21.4)	26	(23.9)	1	(5.9)	0.093
**Activity**							
Transport							
Car	5	(4.0)	5	(4.6)	0	(0.0)	
Motorbike	6	(4.8)	6	(5.5)	0	(0.0)	
Bicycle	6	(4.8)	5	(4.6)	1	(5.9)	
Pedestrian	6	(4.8)	5	(4.6)	1	(5.9)	
Agricultural and forestry vehicles	2	(1.6)	2	(1.8)	0	(0.0)	
Other means of transport	1	(0.8)	1	(0.9)	0	(0.0)	0.870
Flying	2	(1.6)	2	(1.8)	0	(0.0)	0.573
Watersport							
Swimming	15	(11.9)	10	(9.2)	5	(29.4)	
Canoe/kayak (< 3p.)	1	(0.8)	1	(0.9)	0	(0.0)	0.054
Summersport							
Hiking	3	(2.4)	2	(1.8)	1	(5.9)	
Mountain biking	1	(0.8)	1	(0.9)	0	(0.0)	
Alpine touring	1	(0.8)	1	(0.9)	0	(0.0)	
Climbing	2	(1.6)	2	(1.8)	0	(0.0)	
Other summer sport	3	(2.4)	0	(0.0)	3	(17.6)	0.001
Wintersport							
Skiing/Carving	6	(4.8)	5	(4.6)	1	(5.9)	
Other winter sports	2	(1.6)	2	(1.8)	0	(0.0)	0.834
Work							
In agriculture	2	(1.6)	2	(1.8)	0	(0.0)	
In forestry	2	(1.6)	2	(1.8)	0	(0.0)	0.725
Other							
No other activity	66	(52.4)	54	(49.5)	12	(70.6)	
In and around the house	41	(32.5)	37	(33.9)	4	(23.5)	
Horse riding	2	(1.6)	2	(1.8)	0	(0.0)	
Relocation (secondary deployment)	1	(0.8)	1	(0.9)	0	(0.0)	
No activity or unknown	16	(12.7)	15	(13.8)	1	(5.9)	0.571
**Aetiology detail**							
**Non-traumatic**	69	(54.8)	54	(49.5)	15	(88.2)	0.003
Asphyxia—total	35	(27.8)	29	(26.6)	6	(35.3)	0.457
Asphyxia—drowning	17	(13.5)	13	(11.9)	4	(23.5)	0.193
Asphyxia—aspiration	5	(4.0)	5	(4.6)	0	(0.0)	0.368
Asphyxia—strangulation	7	(5.6)	7	(6.4)	0	(0.0)	0.282
Asphyxia—burial	6	(4.8)	4	(3.7)	2	(11.8)	0.145
Other pulmonary	7	(5.6)	7	(6.4)	0	(0.0)	0.282
Cardiac	18	(14.3)	10	(9.2)	8	(47.1)	< 0.001
SIDS	0	[0.0]	0	[0.0]	0	[0.0]	-
Anaphylactic	2	(1.6)	2	(1.8)	0	(0.0)	0.573
Intoxication	0	[0.0]	0	[0.0]	0	[0.0]	-
Non-traumatic, reason unknown	6	(4.8)	5	(4.6)	1	(5.9)	0.816
Other non-traumatic	1	(0.8)	1	(0.9)	0	(0.0)	0.692
**Traumatic**	57	(45.2)	55	(50.5)	2	(11.8)	0.003
Traffic accident	20	(15.9)	20	(18.3)	0	(0.0)	0.054
Fall from a height	20	(15.9)	18	(16.5)	2	(11.8)	0.618
Burning/scalding	3	(2.4)	3	(2.8)	0	(0.0)	0.489
Other traumatic	14	(11.1)	14	(12.8)	0	(0.0)	0.117
**First rhythm**							
**Non-shockable**	109	(86.5)	100	(91.7)	9	(52.9)	< 0.001
Pulseless Electrical Activity	19	(15.1)	18	(16.5)	1	(5.9)	0.255
Asystole	67	(53.2)	64	(58.7)	3	(17.6)	0.002
**Shockable initial rhythm**	17	(13.5)	9	(8.3)	8	(47.1)	< 0.001
Pulseless Ventricular Tachycardia	1	(0.8)	1	(0.9)	0	(0.0)	0.692
Pulseless Ventricular Fibrillation	16	(12.7)	8	(7.3)	8	(47.1)	< 0.001
Normal Sinus rhythm / ROSC	2	(1.6)	0	(0.0)	2	(11.8)	< 0.001
Unknown	6	(4.8)	3	(2.8)	3	(17.6)	0.007
No measures taken (obviously dead)	15	(11.9)	15	(13.8)	0	(0.0)	0.103
**Injuries**							
Traumatic brain injury	41	(32.5)	39	(35.8)	2	(11.8)	0.049
Chest trauma	21	(16.7)	21	(19.3)	0	(0.0)	0.047
Abdominal trauma	13	(10.3)	13	(11.9)	0	(0.0)	0.133
Pelvic trauma	5	(4.0)	5	(4.6)	0	(0.0)	0.368
Upper extremity trauma	0	[0.0]	0	[0.0]	0	[0.0]	-
Lower extremity trauma	9	(7.1)	9	(8.3)	0	(0.0)	0.219
**Outcome**							
Favor. neurol. outcome, 30d (CPC 1/2)	17	(13.5)	0	(0.0)	17	(100.0)	< 0.001

Depending on normality testing (Shapiro Wilk) median (IQR) respectively mean (SD) are shown for continuous variables, *p*-values obtained by Wilcoxon rank sum test respectively unpaired T-test. Categorical variables are shown with number (%) in each category, *p*-values obtained by Chi-squared test

*Abbreviations*: *CPC* Cerebral Performance Category, *ROSC* Return of Spontaneous Circulation, *SIDS* Sudden Infant Death Syndrome

## Geographical impact on outcomes

Geographical location further influences outcomes, with urban and remote settings exhibiting pronounced differences. Urban settings typically allowed for faster EMS response, greater access to advanced medical resources, and higher bystander CPR rates. In contrast, remote areas faced prolonged response times and limited resources, leading to lower survival and neurological outcomes. HEMS teams in Switzerland were generally able to reach any remote location in a reasonable time, averaging 18 min for arrival, although pre-arrival interventions by ground bound EMS proved crucial in managing initial care [8, 9]. In our cohort many of the drowning accidents happened in swimming pools (46.9%, *n*= 30) with a higher survival rate compared to unsupervised drowning accidents in natural waters in rural areas, which often had long down-times due to recovery issues. Remote areas are more frequently associated with high-risk recreational activities, leading to an increased incidence of traumatic accidents, which generally have lower survival rates [7].

## Conclusions

The age-specific aetiology and outcomes in paediatric OHCA emphasize the need for tailored approaches in resuscitation and post-resuscitation care. Infants are especially susceptible to hypoxia-related arrests, while preschool-aged children are most at risk for drowning-related incidents, where early intervention significantly improves neurological outcomes. In school-aged and adolescent children, the high rate of traumatic cardiac arrests necessitates further development of paediatric-focused guidelines for traumatic cardiac arrest management.

Enhancing public awareness, expanding CPR training with a focus on children, and optimizing EMS infrastructure are vital, particularly in rural areas. Recognizing age-specific risks and implementing timely interventions aligned with the causes of arrest can ultimately improve survival and neurological outcomes for paediatric patients experiencing OHCA.

In addition, preventive strategies such as promoting safe sleep practices for infants, ensuring vigilant water safety and supervision for toddlers and preschoolers, and enforcing protective measures in sports and traffic settings for older children are essential. Public health campaigns targeting caregivers and schools can play a pivotal role in reducing the incidence of preventable cardiac arrests among children.

Sincerely,

Deliah Bockemuehl.

## Data Availability

The presented data in the manuscript is available from the authors with a reasonable request and after permission of the responsible ethical committee due to Swiss law.

## Abbreviations

CPC: Cerebral Performance Category
CPR: Cardiopulmonary Resuscitation
EMS: Emergency Medical Service
HEMS: Helicopter Emergency Medical Service
OHCA: Out-of-Hospital Cardiac Arrest
ROSC: Return of Spontaneous Circulation
SIDS: Sudden Infant Death Syndrome
TCA: Traumatic Cardiac Arrest
